# AMPA Receptors in Synaptic Plasticity, Memory Function, and Brain Diseases

**DOI:** 10.1007/s10571-024-01529-7

**Published:** 2025-01-22

**Authors:** Cristina A. Muñoz de León-López, Marta Carretero-Rey, Zafar U. Khan

**Affiliations:** 1 Laboratory of Neurobiology, Centro de Investigaciones Medico Sanitarias (CIMES), University of Malaga, Calle Marqués de Beccaria, 3, Campus Teatinos s/n, 29010 Malaga, Spain; 2 Department of Medicine, Faculty of Medicine, University of Malaga, Campus Teatinos s/n, Malaga, Spain; 3 CIBERNED, Institute of Health Carlos III, Madrid, Spain

**Keywords:** AMPA receptors, Trafficking, LTP and LTD, Homeostatic plasticity, Memory, Aging and neurological diseases

## Abstract

Tetrameric AMPA-type ionotropic glutamate receptors are primary transducers of fast excitatory synaptic transmission in the central nervous system, and their properties and abundance at the synaptic surface are crucial determinants of synaptic efficacy in neuronal communication across the brain. The induction of long-term potentiation (LTP) leads to the insertion of GluA1-containing AMPA receptors at the synaptic surface, whereas during long-term depression (LTD), these receptors are internalized into the cytoplasm of the spine. Disruptions in the trafficking of AMPA receptors to and from the synaptic surface attenuate both forms of synaptic plasticity. Homeostatic scaling up and scaling down, which are additional types of plasticity similar to LTP and LTD, are also regulated by the insertion and removal of GluA1-containing AMPA receptors from the synaptic surface. The trafficking of AMPA receptors is an intricate process assisted by various proteins. Furthermore, AMPA receptors are critical for the formation and consolidation of various types of memory, and alterations in their function are intimately associated with cognitive dysfunction in aging and several neurological and psychiatric diseases. In this review, we will provide an overview of the current understanding of how AMPA receptors regulate various forms of synaptic plasticity, their contribution to memory functions, and their role in aging and brain diseases.

## Introduction

**Fig. 1:**
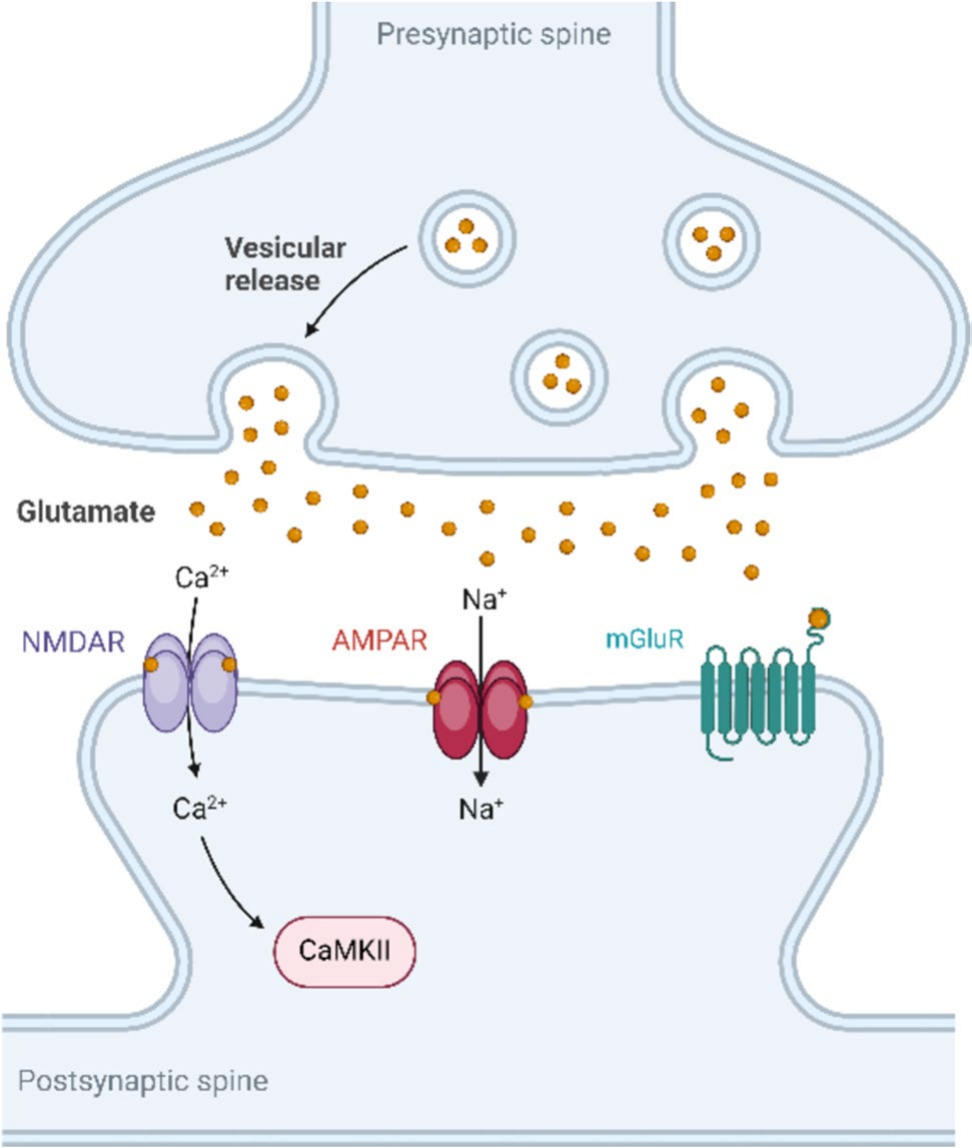
Glutamate receptors at the synaptic surface. A stimulus-mediated glutamate release activates ionotropic AMPA and NMDA receptors, as well as G-protein-coupled mGluRs. Activation of AMPA and NMDA receptors leads to the influx of Na^+^ and Ca^2+^, respectively. The influx of Ca^2+^ activates CaMKII, which is essential for the phosphorylation of AMPA receptors and the induction of synaptic potentiation. In contrast, activation of mGluRs triggers a signaling cascade through second messengers

Glutamate (or glutamic acid) is considered the most abundant excitatory neurotransmitter in the brain. The release of glutamate during neurotransmission activates glutamate receptors in the postsynaptic neurons and regulates a variety of brain functions. There are two types of glutamate receptors: the G-protein-coupled receptor family and the ion channel family. The G-protein-coupled receptor family of glutamate receptors is known as metabotropic glutamate receptors (mGluRs). However, the ion channel family of glutamate receptors, which are also known as ionotropic glutamate receptors (iGluRs), is composed of four different subfamilies: alpha-amino-3-hydroxy-5-methyl-4-isoxazolepropionic acid (AMPA), delta, kainate, and N-methyl-D-aspartate (NMDA) (Reiner and Levitz 2018) (Fig. 1). mGluRs act in concert with G-proteins to induce second messenger pathways and trigger a cascade of biochemical reactions. These types of glutamate receptors produce slow responses and contribute to long-lasting changes in synaptic function (Scheefhals and MacGillavry 2018). In contrast, iGluRs do not require G-proteins. Instead, they assemble into tetramers to form ion channels, and the binding of neurotransmitters directly opens the channel, allowing the flow of Na^+^ and K^+^ ions (Reiner and Levitz 2018). In addition, some subtypes, such as NMDA receptors, as well as certain AMPA and kainate receptors, also allow the flow of Ca^2^⁺ ions. In the mammalian central nervous system (CNS), iGluRs are responsible for rapid excitatory transmission, capable of transmitting excitatory signals to postsynaptic neurons within milliseconds (Scheefhals and MacGillavry 2018). This process generates a synaptic current that is critical for brain functions.

AMPA receptors, part of the iGluRs family, are the primary transducers of fast excitatory synaptic transmission in the mammalian brain and are crucial for plasticity, memory, and synaptic transmission (Hansen et al. 2021; Huganir and Nicoll 2013). The AMPA receptors consist of four subunits: GluA1, GluA2, GluA3 and GluA4 (Keinänen et al. 1990; Bennett et al. 1996). In the adult brain, AMPA receptors typically exist in a heterotetrameric form, primarily composed of two dimers made up of combinations of GluA1, GluA2, or GluA3 subunits. GluA4 is expressed during early developmental stages (Zhu et al. 2000), but in the adult brain, it is absent (Schwenk et al. 2014; Gersdorff and Borst 2002; Pelkey et al. 2015). The functional tetramers of these subunits are assembled within the endoplasmic reticulum and then transported via endosomes to the synaptic surface, where they form ion channels (Greger et al. 2017; Gan et al. 2015). As each subunit of this receptor contributes differently to channel kinetics, ion selectivity, and receptor trafficking, heteromerization of these subunits adds considerable diversity to AMPA receptor functions. AMPA receptor functions are regulated through a complex signaling process that depends not only on ion channel pore formation but also on various auxiliary components (Greger et al. 2017; Kamalova and Nakagawa 2021). In fact, these auxiliary components help determine the basic features of AMPA receptor gating, channel conductance, trafficking, and retention at the synaptic surface (Greger et al. 2017). Additionally, trafficking, synapse retention, and single-channel conductance are also regulated by subunit-specific post-translational modifications, such as phosphorylation (Diering and Huganir 2018).

## AMPA Receptors and Synaptic Plasticity

### AMPA Receptors in Hebbian Forms of Plasticity

**Fig. 2:**
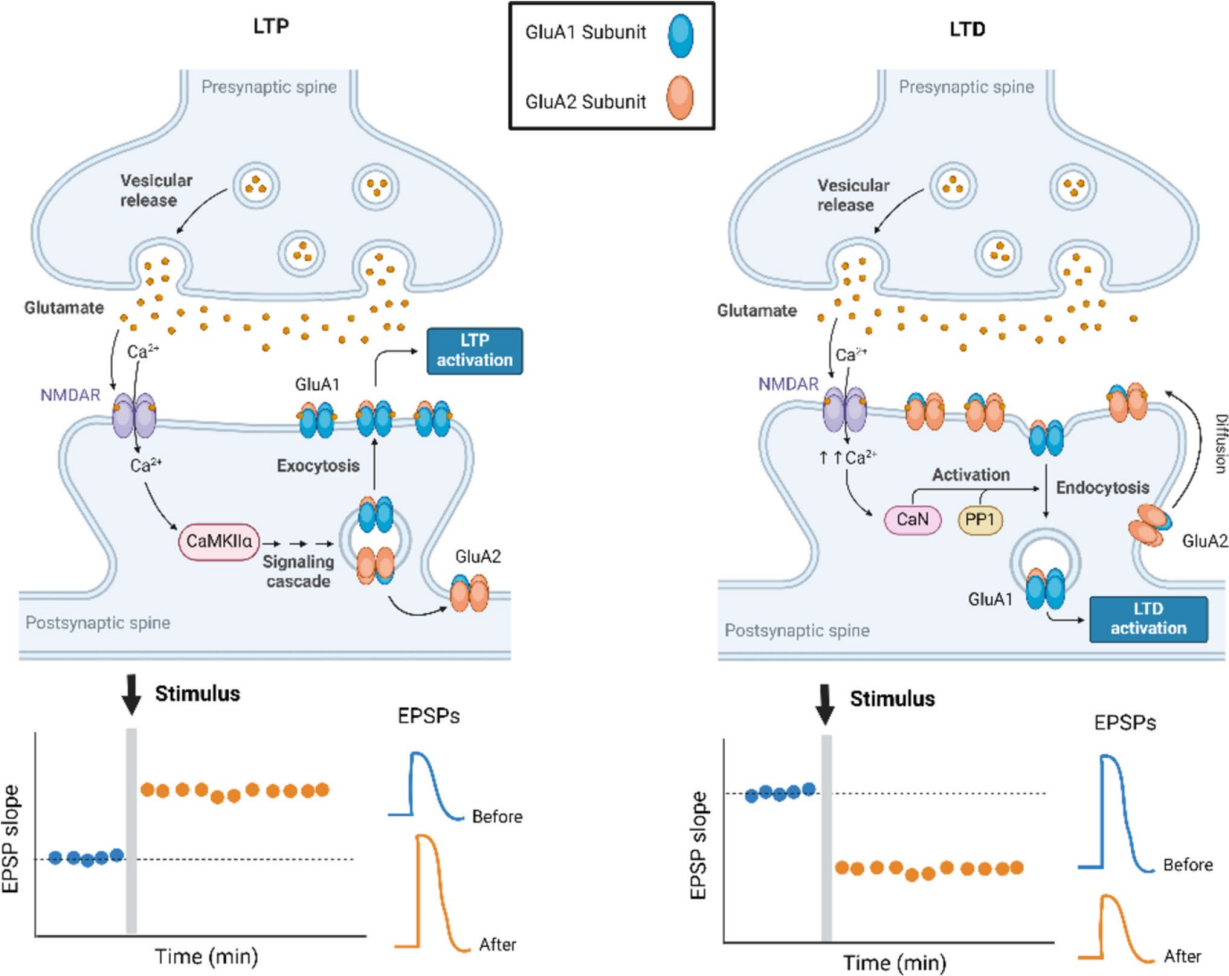
AMPA receptors in LTP and LTD. During LTP (left), GluA1-containing AMPA receptors are inserted into the synaptic surface to maintain synaptic strength, whereas during LTD (right), GluA1-containing AMPA receptors are internalized, leading to synaptic depression. *CaN* calcineurin; *PP1* protein phosphatase 1

AMPA receptors are constantly trafficked to and from the synaptic surface (Chater and Goda 2022). It has been shown that they enter and leave the membrane within seconds to minutes and that the functionally relevant form of long-term potentiation (LTP) relies heavily on the membrane incorporation of AMPA receptors (Díaz-Alonso and Nicoll 2021). The induction of LTP causes the insertion of AMPA receptors at the synaptic surface, while long-term depression (LTD) leads to the removal of AMPA receptors (Chater and Goda 2022) (Fig. 2).

#### GluA1-Containing AMPA Receptors in LTP

Induction of LTP causes the rapid incorporation of GluA1-containing AMPA receptors at the synaptic surface of excitatory synapses. However, these receptors are present at the surface only transiently and are eventually replaced by GluA2-containing AMPA receptors (Isaac et al. 2007; Plant et al. 2006; Chater and Goda 2022). Blocking the synaptic incorporation of GluA1-containing AMPA receptors during this time frame prevents the induction of hippocampal LTP (Terashima et al. 2019). After incorporation into the perisynaptic surface, GluA1-containing AMPA receptors diffuse laterally through the membrane and enter the postsynaptic density area, where they interact with postsynaptic density protein 95 (PSD-95) via intermediary transmembrane AMPA receptor regulatory proteins (TARPs). This interaction facilitates their retention at the synaptic surface and helps align them with presynaptic neurotransmitter release sites (Biederer et al. 2017; Chater and Goda 2022). It is believed that the retention of AMPA receptors at the synaptic surface occurs in nanodomains distributed in small columns within the postsynaptic density area. During LTP, additional columns are formed through the reorganization of postsynaptic density area structural proteins (Tang et al. 2016; Sinnen et al. 2017).

**Fig. 3:**
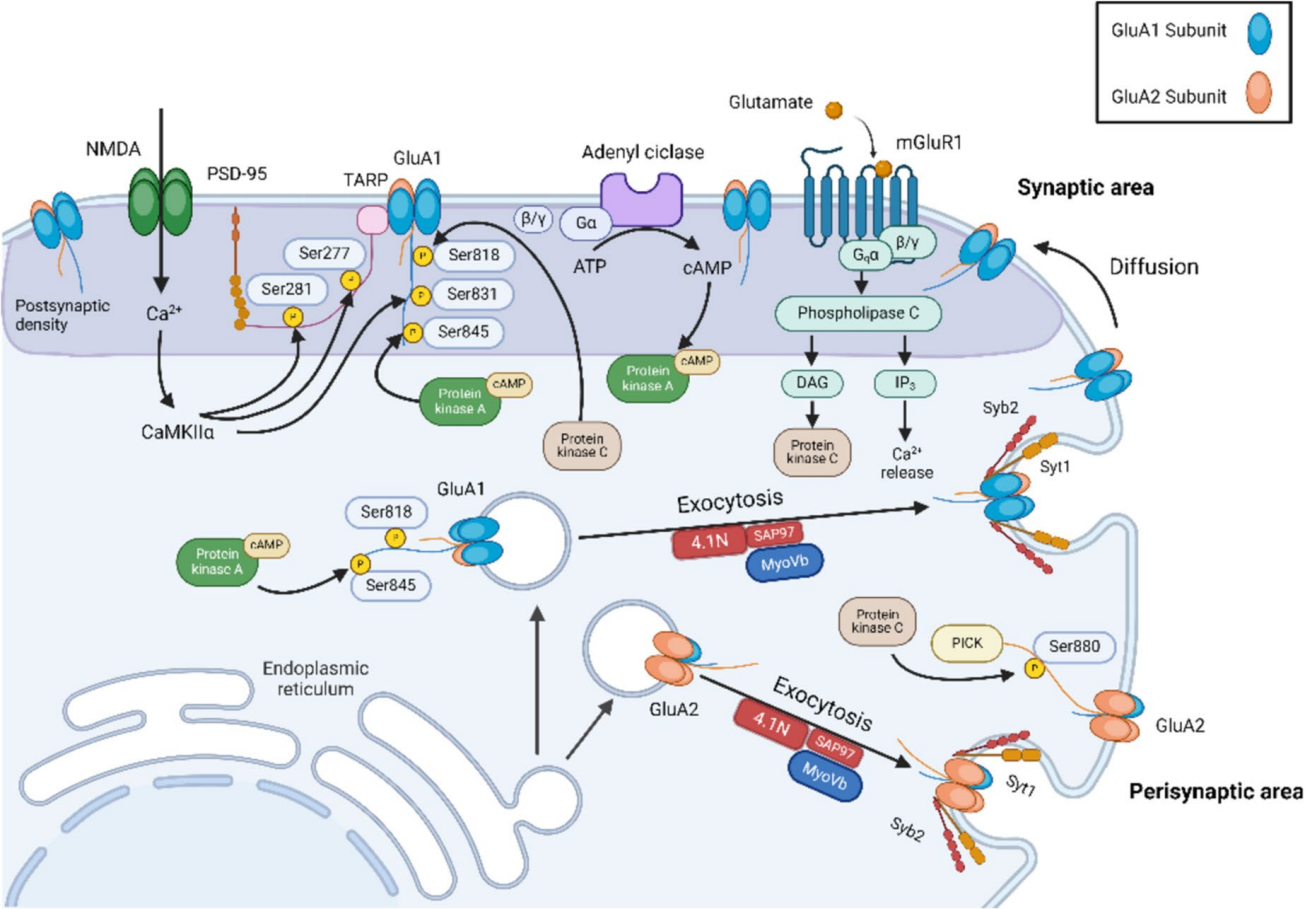
AMPA receptors trafficking during LTP. AMPA receptor subunits are synthesized and assembled in the endoplasmic reticulum, and are then exported to the cytosol, where SAP97 binds to the C-terminus of the GluA1 subunit of AMPA receptors and interacts with the actin-associated protein 4.1N to mediate perisynaptic surface membrane insertion of GluA1-containing AMPA receptors. Once at the perisynaptic membrane, these AMPA receptors laterally diffuse through the surface membrane to reach the postsynaptic density area, where they bind to phosphorylated TARP (pTARP) associated with PSD-95. The binding of GluA1-containing AMPA receptors to the pTARP-(PSD-95) protein complex traps them at the synaptic surface. At the same time, GluA2-containing AMPA receptors are retained at the perisynaptic membrane. NMDA receptor-mediated Ca^2^⁺ influx and activation of CaMKII induce phosphorylation at the S831 site of the GluA1 subunit, promoting LTP and the maintenance of synaptic strength. *cAMP* cyclic AMP; *Syt1* synaptotagmin 1; *Syb2* synaptobrevin 2; *PICK* protein interacting with C Kinase 1; *DAG* diacylglycerol; *TARP* transmembrane AMPA receptor regulatory protein

LTP induction triggers Ca^2+^ influx and the activation of calcium–calmodulin (CaM)-dependent protein kinase II (CaMKII) (Lee et al. 2009). Active CaMKII is then recruited to synapses undergoing LTP and binds to NMDA receptor subunit 2B (GluN2B). The binding of CaMKII is not only a key step in this process but also a prerequisite for information storage during LTP (Yasuda et al. 2022). GluN2B-associated CaMKII augments the functional availability of AMPA receptors at synaptic surface through the phosphorylation of the GluA1 subunit at the S831 site. The phosphorylated GluA1 subunit promotes the maintenance of synaptic strength and LTP (Halt et al. 2012; Olivito et al. 2016; Purkey and Dell’Acqua 2020). In addition to phosphorylating the GluA1 subunit, GluN2B-bound CaMKII also phosphorylates auxiliary proteins such as TARPs (Park et al. 2016; Yasuda et al. 2022). The phosphorylation of the S277 and S281 sites in the cytosolic C-terminal domain of TARPγ8 has been implicated in LTP (Park et al. 2016). These phosphorylations diminish the association of TARPγ8 with the synaptic membrane, thereby increasing the availability of its C-terminal domains for binding to the PDZ domains of PSD-95 (Hafner et al. 2015). This activity facilitates the trapping of GluA1-containing AMPA receptors by forming GluA1-TARP-(PSD-95) complex at the synaptic surface. Therefore, the insertion and stabilization of GluA1-containing AMPA receptors at the synaptic surface facilitate the increase in single-channel conductance and the strengthening of synaptic transmission during LTP (Opazo et al. 2010; Purkey and Dell’Acqua 2020; Yasuda et al. 2022) (Fig. 3).

In addition to the phosphorylation of the S831 site in the GluA1 subunit, which increases single-channel conductance and is critical for the maintenance of synaptic strength (Summers et al. 2019; Diering and Huganir 2018), there are two other phosphorylation sites in the GluA1 subunit: S845 and S818 (Purkey and Dell’Acqua 2020). In contrast, phosphorylation of the GluA1 subunit at the S845 site is mediated by protein kinase A (PKA). This phosphorylation increases channel open probability and peak current amplitude and is essential for the synaptic surface recruitment of GluA1-containing AMPA receptors during the initial stages of potentiation (Yang et al. 2010; Purkey and Dell’Acqua 2020). The phosphorylation of the S818 site on the GluA1 subunit is driven by protein kinase C (PKC). This phosphorylation increases single-channel conductance and promotes the exocytosis and synaptic incorporation of GluA1-containing AMPA receptors (Diering and Huganir 2018).

#### GluA1-Containing AMPA Receptors in LTD

**Fig. 4:**
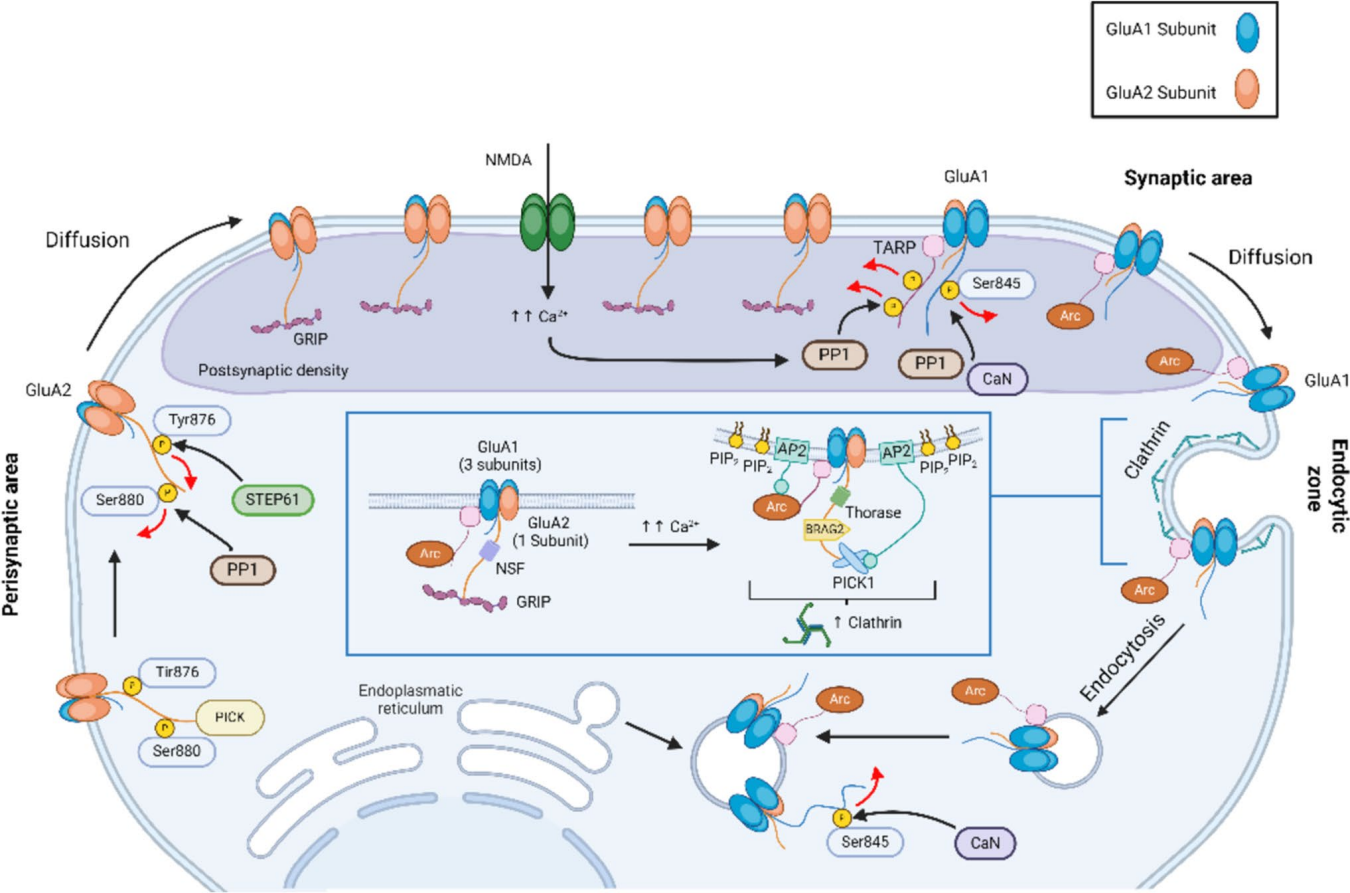
AMPA receptors trafficking during LTD. Phosphatase PP1-mediated dephosphorylation of pTARP disrupts its interaction with PSD-95, freeing the trapped GluA1-containing AMPA receptors. The liberated GluA1 subunits of AMPA receptors are then dephosphorylated at the S845 site and the dephosphorylated TARP binds with Arc, forming the Arc-TARP-AMPA receptor protein complex. This protein complex diffuses through the surface membrane toward the endocytic zone, where Arc and the GluA2 subunit of GluA1-containing AMPA receptors, via PICK1, interact with the AP2 complex for clathrin-mediated endocytosis (inset). *TARP* transmembrane AMPA receptor regulatory protein; *PP1* protein phosphatase 1; *CaN* calcineurin; *NSF* N-ethylmaleimide-sensitive fusion protein; *GRIP* glutamate receptor interacting protein 1; *AP2* assembly polypeptide 2 complex; *PICK1* protein interacting with C Kinase 1; *Arc* Activity-regulated cytoskeleton-associated protein

In contrast to LTP, where GluA1-containing AMPA receptors are trafficked to the synaptic surface and the incorporation of GluA2-containing AMPA receptors into the perisynaptic surface is halted (Chater and Goda 2022), LTD is characterized by the removal of GluA1-containing AMPA receptors from the synaptic surface (Chater and Goda 2022). The formation of the GluA1-TARP-(PSD-95) protein complex through the binding of TARP with PSD-95 is essential for the stabilization of GluA1-containing AMPA receptors at the synaptic surface (Hanley 2018). Thus, prior to endocytosis, the GluA1 subunits of GluA1-containing AMPA receptors dissociate from TARP-(PSD-95) protein complex (Opazo et al. 2012; Parkinson and Hanley 2018). CaMKII-mediated phosphorylation of TARP at its carboxy-terminal facilitates the binding of TARP with PSD-95 (Sumioka et al. 2010). However, the dephosphorylation of the same sites in TARP by protein phosphatase 1 (PP1) disrupts the interaction between PSD-95 and TARP, leading to the release of GluA1 subunits (Sumioka et al. 2010; Tomita et al. 2005). The GluA1 subunits of AMPA receptors are then dephosphorylated at the S845 site, a process that promotes the endocytosis of the receptor (Sanderson et al. 2016). Blockage of dephosphorylation at this site in GluA1 subunits interrupts endocytosis and attenuates LTD (Mulkey et al. 1994; Purkey and Dell’Acqua 2020) (Fig. 4).

In contrast to GluA1 subunits,the GluA2 subunits of GluA1-containing AMPA receptors are trapped by binding to the PDZ domains of glutamate receptor-interacting protein 1 (GRIP1) and the TARP-(PSD-95) protein complex (Opazo and Choquet 2011; Hanley 2018). Induction of LTD dephosphorylates TARP, disrupting its interaction with the GluA2 subunit and the TARP-(PSD-95) protein complex. Additionally, LTD induces phosphorylation of the GluA2 subunit at the S880 site of the C-terminal domain by PKCα, which disrupts the binding of the receptor to GRIP1 (Sumioka et al. 2010; Hanley 2018). The GluA2 subunit of the liberated AMPA receptor then binds to protein interacting with C kinase 1 (PICK1) and diffuses through the spine surface membrane to the endocytic zone, where endocytosis is facilitated by interaction with the assembly polypeptide 2 (AP2) complex (Chung et al. 2000; Zhang and Bramham 2021).

#### GluA2-Containing AMPA Receptors in LTP and LTD

Although most studies in the literature underscore the critical role of GluA1-containing AMPA receptors in LTP, GluA2-containing AMPA receptors have also been shown to participate in this process. While GluA1-containing AMPA receptors are rapidly recruited to the synaptic surface during LTP, GluA2-containing AMPA receptors are recruited to the synaptic surface in a constitutive manner and gradually replace GluA1-containing receptors (Shi et al. 2001; Isaac et al. 2007; Plant et al. 2006; Chater and Goda 2022). This replacement of GluA1-containing AMPA receptors with GluA2-containing AMPA receptors following LTP has been suggested as an important component in memory consolidation. Moreover, it has been shown that LTP in the mature hippocampus is mediated by GluA2-containing AMPA receptors (Adesnik and Nicoll 2007). The GluA2 subunit directly interacts with GRIP1/GRIP2 and PICK1 through their PDZ domains, facilitating the trafficking of GluA2-containing AMPA receptors at the synaptic surface (Anggono and Huganir 2012). The mice lacking PICK1 show reductions in hippocampal LTP as well as defects in hippocampus-dependent learning and memory tasks (Makuch et al. 2011; Volk et al. 2010; Chiu et al. 2017). Additionally, disruption of the interaction between GRIP1 and neuronal endosomal protein 21 (NEEP21), a protein involved in GluA2-containing AMPA receptor trafficking, also impairs LTP (Alberi et al. 2005; Steiner et al. 2005) (Figs. 3 and 4).

LTD is primarily triggered by the activation of NMDA and mGlu receptors, both of which induce the internalization of GluA2-containing AMPA receptors from the synaptic surface (Sanderson et al. 2011a). The C-terminal tail of the GluA2 subunit in GluA2-containing AMPA receptors, which interacts with PDZ proteins that facilitate receptor internalization, is necessary and sufficient to drive LTD (Zhou et al. 2018). Dephosphorylation at the Y876 site of the GluA2 subunit by tyrosine phosphatase, as well as phosphorylation at the S880 site by PKCα, triggers the endocytosis of GluA2-containing AMPA receptors (Gladding et al. 2009; Chung et al. 2000). Additionally, activation of Src homology 2 (SH2) domain-containing phosphatase 2 (SHP2) causes dephosphorylation at the Y869 and Y876 sites of GluA2 subunits, resulting in the endocytosis of GluA2-containing AMPA receptors and LTD (Lee et al. 2024). Phosphorylation of GluA2 subunits at the Y876 site has been shown to prevent phosphorylation at the S880 site and block LTD (Kohda et al. 2013). Therefore, dephosphorylation at Y876 and phosphorylation at S880 in the GluA2 subunit appear to be required for the dissociation of GluA2-containing AMPA receptors from the synaptic surface and subsequent binding to PICK1. Phosphorylation of PICK1 at the S416 site by glycogen synthase kinase 3β (GSK3β) is essential for its interaction with the GluA2 subunit, a critical step in the endocytosis of GluA2-containing AMPA receptors during LTD (Yagishita et al. 2015). GluA2 subunit directly binds to the AP2 complex, which is involved in the clathrin-mediated endocytosis of GluA2-containing AMPA receptors, and this interaction is required for LTD in the hippocampus (Lee et al. 2002; Kastning et al. 2007). These internalized receptors are stored intracellularly in endosomes and recycled to the synaptic surface during LTP; however, during LTD, they are directed to lysosomes for degradation (Hanley 2010; Carroll et al. 2001).

#### Ca^2+^-Permeable AMPA Receptors in LTP and LTD

GluA2-lacking AMPA receptors are Ca^2+^-permeable and are primarily composed of GluA1 subunits, forming either heteromers or homomers (Plant et al. 2006; Chater and Goda 2022). Although Ca^2+^-permeable AMPA receptors are expressed in small amounts in the hippocampus, several studies have shown that LTP induction transiently increases their synaptic surface localization, which contributes to synaptic strengthening (Guire et al. 2008; He et al. 2009; Plant et al. 2006; Yang et al. 2010). Furthermore, synaptic surface localization of Ca^2+^-permeable AMPA receptors is dynamically regulated during the expression of LTP. An increase in the synaptic incorporation of Ca^2+^-permeable AMPA receptors was also observed during LTD induction in hippocampal CA1 neurons (Sanderson et al. 2016). Continuous application of the antagonist NASPM significantly reduced LTD expression (Sanderson et al. 2016). However, this increase in Ca^2+^-permeable AMPA receptors at the synaptic surface is transient, as it could not be detected after LTD induction was completed (Sanderson et al. 2016).

PKA-mediated phosphorylation at the S845 site of the GluA1 subunit is important for the localization of Ca^2+^-permeable AMPA receptors to the synaptic surface during LTP, whereas dephosphorylation of the same S845 site by the Ca^2+^ -dependent phosphatase calcineurin (CaN) is essential for the endocytosis of Ca^2+^-permeable AMPA receptors during LTD (He et al. 2009; Sanderson et al. 2016). PKA and CaN are anchored at the synaptic membrane through A-kinase anchoring protein (AKAP) 150 (AKAP150), and they interact with GluA1 subunits that are associated with synapse-associated protein 97 (SAP97) (Colledge et al. 2000; Sanderson et al. 2012). Another study found that phosphorylation of S831 site in GluA1 by AKAP79-anchored PKC promotes the assembly and synaptic incorporation of Ca^2+^-permeable AMPA receptors (Summers et al. 2019).

In addition to GluA2-lacking AMPA receptors, neuronal Ca^2+^ permeability is regulated by mRNA editing at the Q/R site in the GluA2 subunit of GluA2-containing AMPA receptors (Pachernegg et al. 2015). Q/R site editing in the GluA2 subunit replaces glutamine (Q) with arginine (R) within the ion channel pore, thereby blocking receptor-mediated Ca^2+^ entry into neurons (Kask et al. 1998; Kuner et al. 2001). In a healthy brain, the majority of AMPA receptors contain the edited GluA2(R) isoform rather than the unedited GluA2(Q) isoform (Wright et al. 2023). Intact Q/R editing activity is essential for normal brain function and defects in this process have been linked to neurological dysfunctions (Feldmeyer et al. 1999; Konen et al. 2020). Mutant mice deficient in Q/R site editing exhibit impairments in memory and deficits in dendritic architecture; however, LTP in hippocampal pyramidal neurons and CA3-CA1 circuits remains unchanged (Feldmeyer et al. 1999; Konen et al. 2020; Shimshek et al. 2006).

#### GluA3-Containing AMPA Receptors in LTP and LTD

GluA3 knockout has no effect on LTD in the mouse hippocampus; however, these knockout mice show enhanced LTP (Meng et al. 2003). In contrast, LTP is absent in the cortico-amygdala pathway in GluA3 knockout mice (Humeau et al. 2007). Moreover, LTP at the parallel fiber-to-Purkinje cell synapse in the cerebellum depends on GluA3-containing AMPA receptors and not on GluA1-containing AMPA receptors (Gutierrez-Castellanos et al. 2017). In hippocampal CA1 pyramidal neurons, the GluA3-containing AMPA receptors are in a low-conductance state under basal conditions and contribute minimally to synaptic currents. However, an increase in intracellular cAMP levels induces a shift in GluA3-containing AMPA receptor channels to a high-conductance state, leading to synaptic potentiation (Renner et al. 2017). This cAMP-driven synaptic potentiation is mediated by β-adrenergic receptor-activated PKA and the GTPase Ras. These findings reveal a novel form of plasticity at CA1 synapses that is mediated by the activation of GluA3-containing AMPA receptors.

#### GluA4-Containing AMPA Receptors in Plasticity and Neurotransmission

GluA4-containing AMPA receptors are expressed transiently during early postnatal development and then decline, becoming almost absent in adults (Zhu et al. 2000). In the hippocampus, spontaneous activity is sufficient to selectively traffic GluA4-containing AMPA receptors to the synaptic surface and induce enhanced synaptic potentiation (Zhu et al. 2000). Phosphorylation at the S842 site of GluA4-containing AMPA receptors by either PKA or PKC is essential for the insertion of these receptors at the synaptic surface (Zheng and Keifer 2009; Gomes et al. 2007). The activation of PKA at immature synapses leads to robust potentiation through the synaptic surface insertion of GluA4-containing AMPA receptors (Luchkina et al. 2014). Furthermore, the fast kinetics of GluA4-containing AMPA receptors assist in the processing of auditory information at the calyx of Held synapse and cause faster AMPA-receptor-mediated excitatory postsynaptic currents (EPSCs) (Gersdorff and Borst 2002) and GluA4 knockout significantly slows the time course of EPSCs (Yang et al. 2011). Additionally, GluA4-mediated plasticity contributes to the synchronization of CA3-CA1 neuronal populations in the hippocampus during development (Atanasova et al. 2019).

### AMPA Receptors in Homeostatic Plasticity

LTP and LTD are examples of Hebbian forms of synaptic plasticity that operate locally at individual synapses to encode specific information. In contrast, homeostatic plasticity refers to the ability of neurons to prevent neuronal networks from becoming either silent or hyperactive by engaging in homeostatic scaling up or down of synaptic strength, respectively (Turrigiano 2012; Turrigiano et al. 1998; Pozo and Goda 2010). Therefore, homeostatic plasticity is a mechanism that stabilizes entire neuronal network activity by adjusting synaptic strength, ensuring normal brain function (Antoine et al. 2019).

#### Homeostatic Scaling Up

Similar to Hebbian forms of synaptic plasticity, homeostatic scaling alters synaptic strength by modulating the levels of AMPA receptors at the synaptic surface. The induction of homeostatic scaling up, whether through treatment of neuronal cultures with tetrodotoxin or visual deprivation in mice, increases GluA1-containing AMPA receptors at the synaptic surface in the form of GluA1 homomers and GluA1/GluA2 heteromers (Diering et al. 2014; Soares et al. 2013; Goel et al. 2011). Homeostatic scaling up involves an increase in phosphorylation at the S845 site of the GluA1 subunit (Kim and Ziff 2014; Goel et al. 2011). This increase in GluA1 subunit phosphorylation is facilitated by the coordinated activity of PKA, recruited by the signaling scaffold protein AKAP5, and the concurrent suppression of the phosphatase CaN activity (Diering et al. 2014).

**Fig. 5:**
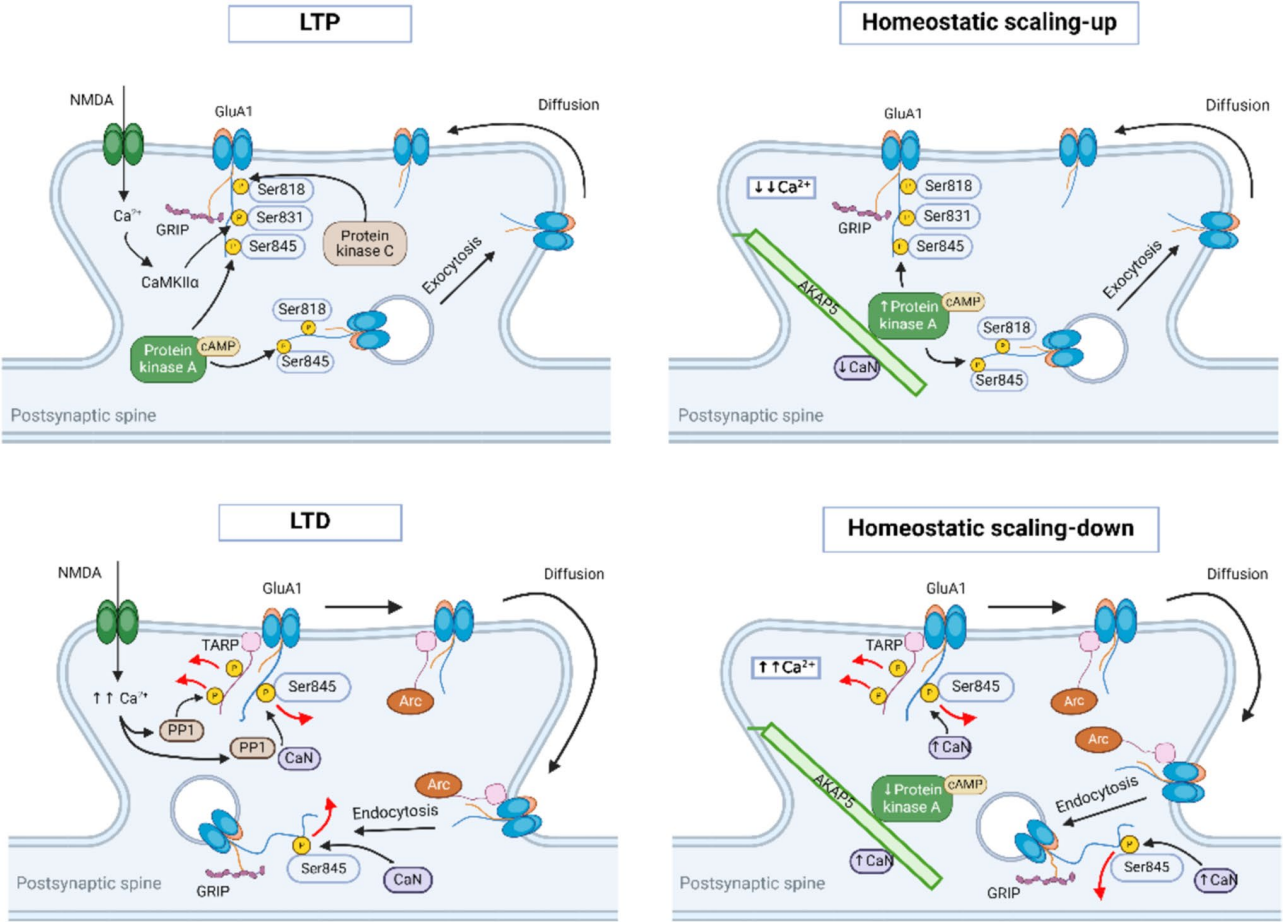
AMPA receptors in Hebbian and homeostatic plasticity. In Hebbian plasticity, synaptic incorporation and CaMKII-mediated phosphorylation at the S831 site of GluA1-containing AMPA receptors lead to synaptic strengthening (LTP, top left), while dephosphorylation at the S845 site and removal of these receptors from the synaptic surface cause synaptic weakening (LTD, bottom left). In contrast, in homeostatic plasticity, phosphorylation of the S845 site by PKA, associated with AKAP5, results in receptor accumulation at the synaptic surface (scaling-up, top right), whereas reduced PKA activity and S845 dephosphorylation by calcineurin (CaN) decrease receptor presence at the synaptic surface (scaling-down, bottom right). *CaN* calcineurin; *TARP* transmembrane AMPA receptor regulatory protein; *GRIP* glutamate receptor interacting protein 1; *Arc* activity-regulated cytoskeleton-associated protein; *cAMP* cyclic AMP; *PP1* protein phosphatase 1

In addition to GluA1, GluA2 subunits also play an essential role in homeostatic scaling up. shRNA-mediated knockdown of the GluA2 subunit blocks homeostatic scaling up, a blockage that can be rescued by expressing shRNA-resistant wild-type GluA2 (Gainey et al. 2009). Homeostatic scaling up is facilitated by the targeting of GRIP1, a binding partner of the GluA2 subunit, to the postsynaptic density area, which assists in the recruitment of GluA2-containing AMPA receptors to the synaptic surface (Tan et al. 2015). Increased phosphorylation at the Y876 site in GluA2 subunits enhances binding with GRIP1 and leads to the accumulation of GluA2-containing AMPA receptors at the synaptic surface (Yong et al. 2020) (Fig. 5).

#### Homeostatic Scaling Down

During scaling down, the GluA1 subunit of AMPA receptors at the synaptic surface is dephosphorylated at the S845 site (Diering et al. 2017). This decrease in phosphorylation at the S845 site is mediated by reduced PKA activity, which occurs due to the uncoupling of PKA from the AKAP5 scaffold protein, thereby favoring the unphosphorylated state of the GluA1 subunit (Diering et al. 2014). AMPA receptors containing the unphosphorylated form of the GluA1 subunit are then preferentially removed from the synaptic surface. Furthermore, induction of homeostatic scaling down by bicuculline, a GABA_A_ receptor antagonist, promotes ubiquitination of the GluA1 subunit at the K868 site, a post-translational modification that may facilitate the internalization of GluA1-containing AMPA receptors from the synaptic surface (Widagdo et al. 2017) (Fig. 5).

Additionally, GRIP1 protein is upregulated during scaling down but remains in intracellular compartments, where it may sequester GluA2-dominant, GluA1-containing AMPA receptors away from the synapse (Tan et al. 2015). Bicuculline treatment also stimulates ubiquitination of GluA2 at the K882 site (Widagdo et al. 2015), which is required for homeostatic scaling down (Scudder et al. 2014).

### Proteins Implicated in AMPA Receptor Trafficking During LTP and LTD

Trafficking of AMPA receptors to and from the synaptic surface is a complex process that depends on interactions between a variety of proteins. Here, we will highlight some of these critical proteins that have a profound effect on synaptic plasticity and have not been largely discussed in the previous sections.

#### Exocytosis of AMPA Receptors During LTP

##### Myosin Vb

Myosin Vb is implicated in endosomal trafficking, and blocking its function impairs LTP (Wang et al. 2008; Sumi and Harada 2020). Myosin Vb binds to endosomes carrying GluA1-containing AMPA receptors via the GTPase Rab11 and its effector Rab11-family interacting protein 2 (Rab11-FIP2), transporting them to the perisynaptic surface area of the spine. The exocytosis of these receptors to the synaptic surface membrane is mediated by the calcium-sensor synaptic vesicle protein synaptotagmin 1 (Syt1), along with other proteins such as synaptotagmin 7 (Syt7), synaptobrevin 2 (Syb2, also known as VAMP2), and complexin (Wu et al. 2017). Additionally, Myosin Vb facilitates the internalization of endosomes carrying AMPA receptors into the cytosol during LTD stimulation (Sumi and Harada 2020).

##### Protein 4.1N

Protein 4.1N is a scaffold protein that belongs to the larger 4.1 family of proteins and is predominantly expressed in dendritic spines (Walensky et al. 1999). 4.1N interacts with SynCAM1, a synaptic cell adhesion molecule and promotes the recruitment of GluA1-containing AMPA receptors (Hoy et al. 2009). The induction of LTP promotes PKC-mediated phosphorylation at the S818 site of the GluA1 subunit of AMPA receptors. Protein 4.1N then interacts with the phosphorylated form of GluA1-containing AMPA receptors to facilitate their exocytosis to the synaptic surface membrane (Qu et al. 2021). Consequently, knockdown of 4.1N impairs LTP (Lin et al. 2009), and when 4.1N binding is disrupted by deleting the phosphorylation site in the GluA1 subunit, a substantially lower frequency of exocytosis is observed (Bonnet et al. 2023).

##### SAP97

SAP97 is a member of the MAGUK family of proteins and plays a major role in the intracellular trafficking and targeting of AMPA receptors (Fourie et al. 2014). This protein is localized throughout the secretory trafficking pathway and at the postsynaptic density area (Bonnet et al. 2023). The PDZ domain of SAP97 interacts directly with the last four amino acids at the carboxy-terminal of the GluA1 subunit of the AMPA receptor (Cai et al. 2002). SAP97 acts as an adaptor between the GluA1 subunit and Myosin V, facilitating the exocytosis of GluA1-containing AMPA receptors to the perisynaptic surface area of the spine (Wu et al. 2002). Induction of LTP promotes the interaction between SAP97 and the GluA1 subunit, and this interaction is essential for the intracellular trafficking of GluA1-containing AMPA receptors (Bonnet et al. 2023).

#### Endocytosis of GluA1-Containing AMPA Receptors During LTD

##### Arc

Activity-regulated cytoskeleton-associated protein (Arc; also known as Arg3.1) regulates AMPA receptor endocytosis (Zhang and Bramham 2021) and is critical for LTD and homeostatic scaling down (Waung et al. 2008; Korb et al. 2013). Overexpression of Arc leads to the endocytosis of GluA1-containing AMPA receptors and a decrease in synaptic strength (Rial Verde et al. 2006; Kyrke-Smith et al. 2021). LTD induction activates calcium/calmodulin-dependent phosphatase PP1, which dephosphorylates TARP in the GluA1-TARP-(PSD-95) complex. As Arc has a 20-fold higher affinity for dephosphorylated TARP, its binding detaches the GluA1-TARP complex from the synaptic surface at the postsynaptic density area (Tomita et al. 2005). The endocytosis of the liberated GluA1-TARP-Arc complex at the endocytic zone is facilitated by the interaction of Arc with the AP2 complex and endophilin (Zhang et al. 2015; Zhang and Bramham 2021). Disrupting the interaction of Arc with the AP2 complex impairs the internalization of GluA1-containing AMPA receptors and prevents the decline in synaptic strength (DaSilva et al. 2016). In addition, at a later stage of the clathrin-mediated endocytosis process, Arc binds to endophilin and recruits dynamin to the neck of the endocytic vesicle to facilitate membrane fission (Chowdhury et al. 2006; Ferguson and Camilli 2012).

##### AP2 Complex

The AP2 complex is a multimeric protein composed of four adaptins: α, β2, μ2, and σ2. AP2 is an essential component of clathrin-mediated endocytosis, recruiting GluA2-containing AMPA receptors at the endocytic zone (Hanley 2018). Following dissociation from the synaptic surface at the postsynaptic density area, AMPA receptors (coupled to the unphosphorylated form of TARP) laterally diffuse through the membrane to the endocytic zone, where they interact with the AP2 complex clustered at PI(4,5)P2-rich areas. The GluA2 subunit of the AMPA receptor directly binds to the μ2 subunit of the AP2 complex (Lee et al. 2002; Kastning et al. 2007). The interaction between the AP2 complex and the GluA2 subunit of AMPA receptors is required for NMDAR-dependent LTD in the hippocampus (Lee et al. 2002). Additionally, the carboxy-terminal of unphosphorylated TARP can also bind to the μ2 subunit of the AP2 complex (Sumioka et al. 2010; Matsuda et al. 2013). Nine serine residues at the carboxy-terminal of TARP are essential for its binding with AP2, and mutations in these serine residues disrupt LTD (Nomura et al. 2012; Tomita et al. 2005). Since the GluA1 subunit of the AMPA receptor does not bind to the μ2 subunit of the AP2 complex (Kastning et al. 2007), and Ca^2+^-permeable AMPA receptors composed of GluA1 homomers play a crucial role during LTP (Man 2011), the recruitment and endocytosis of these GluA1 homomer AMPA receptors at the endocytic zone may depend on the interaction between TARP and the μ2 subunit of the AP2 complex.

##### PICK1

PICK1 contains PDZ and BAR domains, allowing it to bind to a variety of proteins and lipid molecules. Following the phosphorylation of the GluA2 subunit of the AMPA receptor and its detachment from GRIP1 at the synaptic surface, the PDZ domain of PICK1 binds to the carboxy-terminal of the GluA2 subunit and facilitates its movement toward the endocytic zone. Disruption of the interaction between PICK1 and the GluA2 subunit impairs AMPA receptor internalization and obstructs LTD (Iwakura et al. 2001; Daw et al. 2000). In contrast to the GluA2 subunit of the AMPA receptor and TARP, which bind to the μ2 subunit of the AP2 complex involved in cargo recruitment, PICK1 interacts with the α-adaptin subunit of the AP2 complex through its BAR domain. This interaction aids in clustering GluA2-containing AMPA receptors at the endocytic site (Fiuza et al. 2017). The mutations in critical aspartate residues of PICK1, changing them to alanine, disrupt its binding to the AP2 complex and impede the endocytosis of GluA2-containing AMPA receptors (Fiuza et al. 2017). Apart from binding to the GluA2 subunit of the AMPA receptor and α-adaptin of the AP2 complex, PICK1 also interacts with dynamin, a GTPase protein involved in clathrin-mediated endocytosis, and enhances dynamin polymerization (He et al. 2011; Fiuza et al. 2017). Dynamin forms a helical polymer around the neck of a budding vesicle during endocytosis and mediates membrane fission (Antonny et al. 2016). Disruption of dynamin activity or mutations in the PICK1 gene prevent LTD expression (Wang and Linden 2000; Linden 2012; Steinberg et al. 2006).

## AMPA Receptors in Memory Functions

AMPA receptors are crucial for memory functions. Blockade of AMPA receptor function leads to impairments in the consolidation and extinction of fear memories, as well as in the consolidation and reconsolidation of spatial and recognition memory (Santoyo-Zedillo et al. 2014; Furini et al. 2020).

### Recognition Memory

Recognition memory is the fundamental ability to recognize previously encountered individuals, objects, and events. The object recognition memory (ORM) task is a widely used paradigm for evaluating this type of memory (Brown and Aggleton 2001; Winters et al. 2008). In the hippocampus, AMPA receptors and L-type voltage-dependent calcium channels (L-VDCC) are essential for both the consolidation and reconsolidation of object recognition memory (ORM), whereas CaMKII is specifically required only for the consolidation of ORM (Furini et al. 2020). Studies involving antagonist infusion into the brain have shown that AMPA receptors are essential for encoding, retrieval, consolidation, and reconsolidation of ORM (Winters et al. 2008; Robbins and Murphy 2006; Man et al. 2022). Additionally, blocking the endocytosis of GluA2-containing AMPA receptors disrupts the retrieval of ORM (Cazakoff and Howland 2011). Furthermore, deletion of the GluA1 subunit of the AMPA receptor impairs ORM (Sanderson et al. 2011b). In addition to ORM, AMPA receptors are also involved in social recognition memory (Marcondes et al. 2020) and associative recognition memory (Barker and Warburton 2015).

### Fear Memory

Fear conditioning induces the insertion of GluA1-containing AMPA receptors at the synaptic surface (Matsuo et al. 2008; Nedelescu et al. 2010; Penn et al. 2017), and the inactivation of these synaptic AMPA receptors erases fear memory (Takemoto et al. 2017). It has been shown that Ca^2+^-permeable AMPA receptors, which are either GluA1-dominant heteromers or GluA1 homomers, increase at the synaptic surface within a few minutes following fear conditioning in the lateral amygdala and these receptors remain there for several hours (Hong et al. 2013; Clem and Huganir 2010). However, during consolidation, Ca^2+^-permeable AMPA receptors are replaced by Ca^2+^-impermeable AMPA receptors, which are GluA2-containing AMPA receptors (Hong et al. 2013). The exchange between Ca^2+^-impermeable AMPA receptors and Ca^2+^-permeable AMPA receptors is dependent on AMPA receptor trafficking (Han et al. 2022), and a switch from Ca^2+^-impermeable AMPA receptors to Ca^2+^-permeable AMPA receptors has been linked to the destabilization of the memory trace observed during fear memory retrieval (Hong et al. 2013). Therefore, it has been suggested that the expression of Ca^2+^-permeable AMPA receptors on the synaptic surface is associated with plasticity and a labile state of memory, whereas its replacement by Ca^2+^-impermeable AMPA receptors may promote memory stabilization (Torquatto et al. 2019). Using the GluA1 antagonist NASPM, it was demonstrated that Ca^2+^-permeable AMPA receptor activity in the lateral amygdala and hippocampus is crucial during the initial phase of consolidation, when the memory trace is still unstable, and that blocking this receptor prevented memory stabilization (Torquatto et al. 2019).

### Spatial Memory

Spatial memory is a fundamental cognitive ability that provides the brain with information about the location of objects or events. The deficits in spatial memory are often an early symptom of aging and neurodegenerative diseases and are important factors in assessing functional cognitive disability in the elderly (Lester et al. 2017; Han et al. 2022). GluA1-containing AMPA receptors are crucial for the consolidation of spatial memory (Lee et al. 2003). A reduction in the level of GluA1-containing AMPA receptors in the hippocampus impairs spatial memory learning and leads to a decrease in LTP (Yaka et al. 2007). Additionally, a GluA1 subunit-specific antagonist, NASPM, produces deficits in animal performance on spatial memory tests (Torquatto et al. 2019). Furthermore, GluA1 subunit knockout mice and knock-in mice with mutations that impair phosphorylation at the S831 and S845 residues not only exhibit defective LTP and LTD but also show deficits in spatial memory (Lee et al. 2003; Sanderson et al. 2010). In contrast to the GluA1 subunit, the impairments in both spatial working and spatial reference memories following conditional deletion or knockdown of the GluA2 subunit in mice suggest that the GluA2 subunit is also necessary for these types of spatial memory (Han et al. 2022; Shimshek et al. 2006). A study found that alterations in GluA2 subunit expression were sufficient to induce deficits in spatial reference memory (Han et al. 2022).

## AMPA Receptors in Brain Diseases and Aging

As AMPA receptors are critical for excitatory synaptic transmission in the brain, alterations in these receptor functions have been associated with several neurological and cognitive disorders. Excessive activation of glutamate receptors is thought to be a major cause of excitotoxicity, neurological injury, and cell death, primarily due to excessive glutamate release from axon terminals. However, studies also show that a reduction in GluA2 subunit expression leads to the formation of GluA2-lacking AMPA receptor channels on the plasma membrane, primarily composed of GluA1 subunits, which are Ca^2+^-permeable and facilitate increased Ca^2+^ influx into neurons. This elevated calcium influx induces neuronal overactivation, ultimately resulting in excitotoxicity and cell death (Bennett et al. 1996; Guo and Ma 2021).

### Epilepsy

Epilepsy is characterized by recurrent seizures and occurs due to excessive electrical discharge, leading to a transient imbalance between excitatory and inhibitory neuronal activities (Lih et al. 2023). The studies show that there are increased levels of extracellular glutamate in patients with epilepsy (Sarlo and Holton 2021). In line with this observation, studies in animal models of partial epilepsy have found not only an increase in glutamatergic excitatory activity but also a decrease in GABAergic inhibitory activity (Rogawski et al. 1995). Evidence from both preclinical and clinical studies showing that AMPA receptor antagonists can inhibit seizures further underscores the importance of the glutamatergic system in mediating epilepsy (Barker-Haliski and Steve White 2015; Chang et al. 2015; Celli and Fornai 2021). Gene mutations identified in epileptic patients are associated with defects in the trafficking of AMPA receptors at the synaptic surface (Zhu et al. 2017; Eiro et al. 2023).

Furthermore, overstimulation of glutamate receptors, leading to excessive intracellular calcium concentrations, is a major cause of neuronal cell death in epilepsy (Lorgen et al. 2017). The primary source of increased calcium during this excitotoxicity is the influx through NMDA subtype glutamate receptors (Lorgen et al. 2017). However, following a neurological insult such as an epileptic seizure, the GluA2 AMPA receptor is downregulated (Lorgen et al. 2017). This downregulation increases the likelihood of forming GluA2-lacking, Ca^2+^-permeable AMPA receptors, which may further exacerbate the toxicity of glutamate neurotransmission.

### Amyotrophic Lateral Sclerosis

The core pathology of amyotrophic lateral sclerosis (ALS) involves the progressive degeneration of motor neurons in both the brain and spinal cord, which are responsible for regulating voluntary muscle activities. As these neurons degenerate, the brain’s ability to initiate and control muscle movement is eventually lost (Ravits and La 2009). Although the exact mechanism underlying ALS pathogenesis remains unclear, early biochemical studies have revealed elevated glutamate levels in the cerebrospinal fluid of ALS patients (Shaw et al. 1995). Moreover, an abnormal increase in Ca^2+^-permeable GluA1 AMPA receptors, along with a decrease in Ca^2+^-impermeable GluA2-containing AMPA receptors, suggests that the motor neurons of ALS patients are hyperexcitable (Kawahara and Kwak 2005; Gregory et al. 2020). The GluA2 subunits of AMPA receptors in the adult brain are edited at the Q/R site, changing glutamine (Q) to arginine (R) within the ion channel pore. This Q/R editing renders the receptor calcium-impermeable and is essential for normal GluA2 function (Pachernegg et al. 2015). However, Q/R site editing is compromised in ALS (Hideyama et al. 2012; Yamashita and Kwak 2014) and the studies have shown that motor neurons in ALS models exhibit altered calcium homeostasis (Guatteo et al. 2007). Since AMPA receptors containing unedited GluA2 at the Q/R site are calcium-permeable, inefficient GluA2 Q/R site editing in ALS may lead to neuronal hyperexcitability and excitotoxicity (Prior-González et al. 2023).

### Alzheimer’s Disease

**Fig.6:**
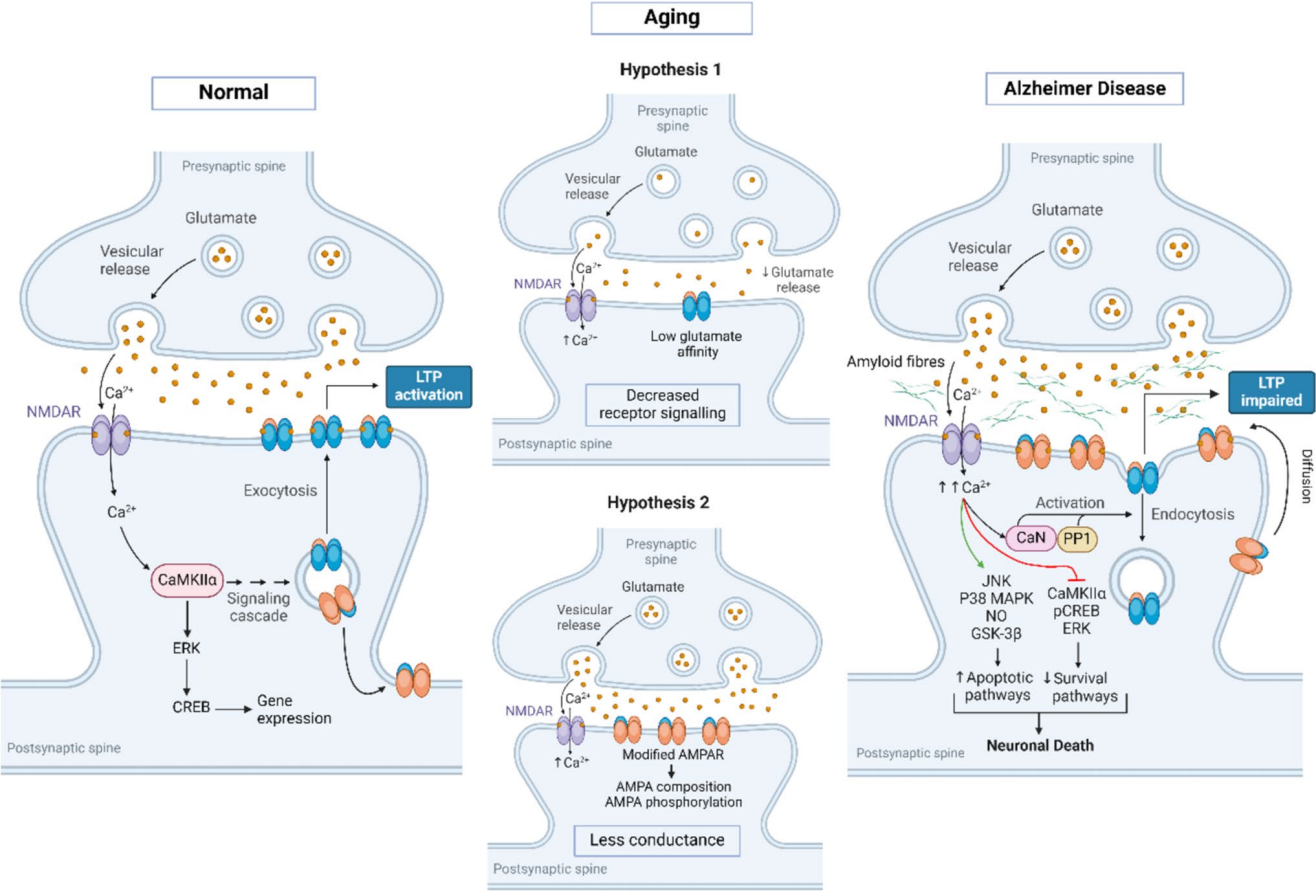
AMPA receptor trafficking in aging and AD. During aging, LTP becomes less robust, an effect thought to be associated with hypofunction in AMPA receptors. There are two views on how this occurs: one suggests an overall decrease in receptor signaling due to a reduced abundance of AMPA receptors at the synaptic surface, while the other proposes that there is reduced conductance because AMPA receptors at the synaptic surface are modified, altering their function. In Alzheimer’s disease, however, Aβ interferes with CaMKII activity, and the lack of CaMKII-mediated phosphorylation of TARP and the GluA1 subunit disrupts the retention of GluA1-containing AMPA receptors at the synaptic surface, leading to an impairment in LTP. *ERK* extracellular signal-regulated kinase; *CREB* cAMP response element-binding protein; *JNK* c-Jun N-terminal kinase; *P38 MAPK* p38 mitogen-activated protein kinase; *NO* nitric oxide; *GSK3b* glycogen synthase kinase 3b; *pCREB* phosphorylated cAMP response element-binding protein

Alzheimer's disease (AD) is a leading cause of age-related dementia (Qu et al. 2021). The deficits in synaptic plasticity, which alter the communication dynamics between neurons, are considered a hallmark of the early etiology of AD (Chakroborty et al. 2019). Additionally, amyloid-β (Aβ) and hyperphosphorylated tau (p-tau) are two distinct aggregates in the brain that play key roles in AD development (Qu et al. 2021). Aβ has been shown to interfere with CaMKII activity, disrupting the phosphorylation-dependent function of GluA1-containing AMPA receptors and impairing LTP and LTD (Gu et al. 2009) (Fig. 6). The post-synaptic protein Bin1, a genetic risk factor for late-onset AD, interacts with the GluA1 subunit of AMPA receptors through Arf6 to regulate the exocytosis of GluA1-containing AMPA receptors. It has been shown that Bin1 is reduced in AD mice, leading to an accumulation of recycling endosomes carrying GluA1-containing AMPA receptors (Schürmann et al. 2020; Qu et al. 2021). In contrast to Aβ, p-tau accumulates in dendritic spines and disrupts AMPA receptor trafficking (Polanco et al. 2018).

### Schizophrenia

Schizophrenia is a complex neuropsychiatric disorder (Joyce and Roiser 2007; Zhang et al. 2021). The binding of TARP with PSD-95 is crucial for trapping GluA1-containing AMPA receptors at the synaptic surface by forming a GluA1-TARP-(PSD-95) protein complex (Hanley 2018). However, in schizophrenia, TARP expression is reduced, which may contribute to lower levels of GluA1-containing AMPA receptors at the synaptic surface (Benesh et al. 2022). Accordingly, fewer GluA1 subunits are found at the synaptic surface in schizophrenia (Benesh et al. 2022). Furthermore, a reduced GluA1/GluA2 AMPA receptor ratio at the synaptic surface and an increased level of GluA1-containing AMPA receptors in endosomes have also been observed in schizophrenia (Benesh et al. 2022). Additionally, mutations in the SHANK gene have been linked to schizophrenia (Peykov et al. 2015; Homann et al. 2016). SHANK is a synaptic scaffolding protein found in the postsynaptic density of excitatory spines, aiding in the recruitment and assembly of multiple postsynaptic molecules (Hayashi et al. 2009). SHANK forms the SHANK-GKAP-PSD95-TARP-AMPA receptor protein complex (Naisbitt et al. 1999; Kim et al. 2001), which is vital for dendritic spine morphology and synaptic plasticity (Sala et al. 2001; Zhang et al. 2021).

### Aging

To date, the research on the role of AMPA receptors in normal aging is limited to a few studies. It has been suggested that there is overall AMPA receptor hypofunction in aged subjects (Jurado 2018). Consistent with this, the studies have shown that LTP in aged animals is less robust and requires stronger stimulation protocols to be elicited (Tombaugh et al. 2002). Additionally, treatment with a positive allosteric modulator of AMPA receptors has been found to restore both LTP and memory in aged animals (Radin et al. 2016). Furthermore, changes in AMPA receptor composition have been observed to contribute to cognitive deficits associated with aging (Hara et al. 2012; Jurado 2018). Accordingly, there are two alternative hypotheses: (a) aging alters presynaptic function, thereby reducing glutamate release and decreasing receptor signaling, and (b) aging causes alterations in AMPA receptor composition and phosphorylation, leading to reduced conductivity (Jurado 2018) (Fig. 6).

## Concluding Remarks

AMPA receptors play a fundamental role in the regulation of Hebbian and homeostatic plasticity, and they are crucial for the formation and consolidation of various types of episodic memory, which is primarily affected in individuals with memory deficits. The insertion of GluA1-containing AMPA receptors at the synaptic surface appears to be a key step in synaptic potentiation and scaling up, while their removal from the synaptic surface is critical for synaptic depression and scaling down. This insertion and removal of GluA1-containing AMPA receptors are governed by a complex trafficking process between the spine cytoplasm and the synaptic surface, assisted by an array of proteins. In contrast to GluA1-containing AMPA receptors, the role of GluA2- and GluA3-containing AMPA receptors in modulating synaptic strength is becoming clearer, but the literature remains limited. Additionally, the precise roles of AMPA receptors with different subunit combinations are still not fully understood. Dysfunction or alterations in AMPA receptors are closely related to cognitive deficits in aging and various neurological and psychiatric diseases. Therefore, continued research on AMPA receptors is essential for understanding how different subunit combinations coordinate various forms of synaptic plasticity, their effects on channel conductance, circuit dynamics, and behavior, and for identifying novel drug targets for developing therapies against memory dysfunctions and neurological diseases.

## Data Availability

No datasets were generated or analysed during the current study.
